# Effect of Pre-Slaughter Practises and Early Post-Mortem Interventions on Sheep Meat Tenderness and Its Impact on Microbial Status

**DOI:** 10.3390/foods11020181

**Published:** 2022-01-11

**Authors:** Carlos Álvarez, Leonard Koolman, Michael Whelan, Aidan Moloney

**Affiliations:** 1 Teagasc Food Research Centre, Department of Food Quality and Sensory Science, D15 DY05 Dublin, Ireland; 2 Teagasc Food Research Centre, Department of Food Safety, D15 DY05 Dublin, Ireland; leonard.koolman@uce.ie; 3 UCD-Centre of Food Safety, School of Public Health, Physiotherapy & Sports Science, University College Dublin, D04 V1W8 Dublin, Ireland; 4 Teagasc Food Research Centre, Meat Technology Ireland, D15 KN3K Dublin, Ireland; michael.whelan@teagasc.ie; 5 Teagasc, Animal & Grassland Research and Innovation Centre, Grange, Dunsany, C15 PW93 Meath, Ireland; Aidan.moloney@teagasc.ie

**Keywords:** sheep meat, electric stimulation, meat aging, eating quality, shelf-life

## Abstract

Tenderness, together with flavour, is the main quality trait that defines consumer acceptance of sheep meat. The factors affecting tenderness can be grouped as those influenced before slaughter, in the early post-mortem intervention and, finally, during the aging period. These factors have been extensively studied with respect to tenderness, but the impact of early post-mortem interventions and subsequent aging on the microbial quality of the final products has not been broadly reviewed to date. In this review, the authors summarize the most recent knowledge on lamb meat tenderness management and how such practices may impact the final meat quality, especially its microbial status. The impacts of pre-slaughter factors (age, sex, diet, genotype and transport) and post-mortem interventions (chilling regime, electrical stimulation, or hanging method), are described and comprehensively discussed.

## 1. Introduction and Scope

Despite global sheep meat consumption amounting to 8.6 M tonnes annually [1], its consumption is inferior to other primary meat sources, such as beef or pork; although, lamb generally has a higher price point than other meats [1]. The eating quality of prime lamb and lamb products is of utmost importance to ensure a good experience for consumers. According to recent reviews [2,3], flavour remains as the main driver for consumer acceptance when purchasing and consuming lamb when tenderness is assured. An understanding of the production and processing factors affecting sheep meat tenderness is, therefore, critical to produce meat products that meet the expectations of diverse consumers. This review discusses the pre-slaughter and carcass processing factors (pre and post rigor) that influence tenderness (and other important eating quality traits) and the microbial status of lamb. Lamb tenderness is influenced by background toughening and the post-mortem toughening and tenderisation phases. Eating quality, including flavour and tenderness, also depends on the production, processing, value-adding and cooking practices carried out by the consumer [4]. In this sense, it is well established that animal sex, age, diet, level of stress and genetic background play a role in determining the final meat quality [3]. Early post-mortem carcass interventions, such as electrical stimulation (ES), aging time, tender-stretching or temperature control after slaughtering are also reviewed. There is a specific focus on the interplay between the effect on eating quality and microbiological effects due to alternate chilling regimes; chilling regimes have to follow the legal requirements in terms of time and temperature to control microbial growth, ensuring that meat is safe to be consumed. 

Therefore, this review aims to provide the readers with the most updated information on the main factors (both ante and post mortem) affecting sheep meat tenderness, and how this may influence the microbial status of the final product. To the best of our knowledge, these two aspects have not been reviewed together before, even though they are tightly interconnected. 

## 2. Pre-Slaughter Influences on Lamb Tenderness

**Figure 1:**
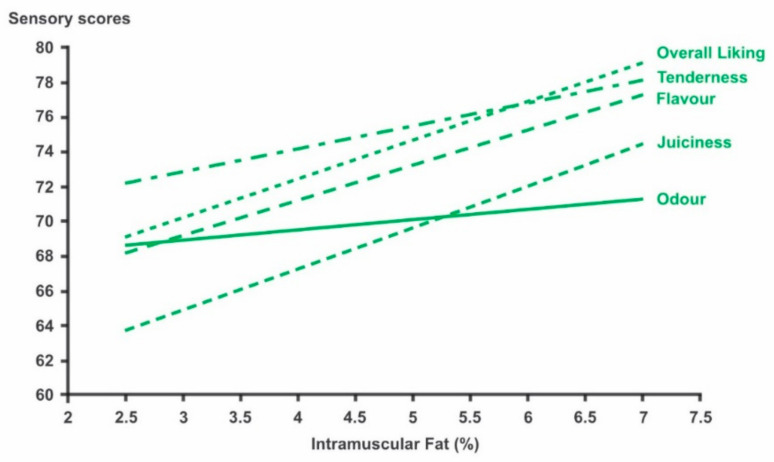
Relationship between IMF (%) and several eating quality traits of the loin muscle of lamb. Adapted from [9].

The pre-slaughter factors that may potentially influence lamb tenderness include: genotype, sex, age at slaughter, diet and associated growth rate, housing/pasture stocking rate, transport to the abattoir and lairage management prior to slaughter. Lamb production systems represent the combined and interactive effects of many of the pre-slaughter factors mentioned above. It is important, therefore, to appreciate that an apparent effect of one factor may be confounded by changes in another factor. For example, an animal consuming a lower energy ration will be older when slaughtered at the same carcass weight as a similar animal consuming a high-energy ration; thus, an apparent effect of ration composition is confounded with the consequent change in age. In addition, pre-slaughter factors may have an indirect effect on tenderness by changing carcass weight, as observed for Awassi ram lambs [5], with consequences for the rate of carcass cooling or intramuscular fat (IMF) concentration, which, itself, can have a direct effect on muscle tenderness (Figure 1). Several reviews of the influence of pre-slaughter factors on lamb tenderness and meat eating quality, in general, have been recently published [3,6,7,8]. This section of the present review is an update of the most pertinent aspects of these reviews with a focus on tenderness and on lamb production where possible.

### 2.1. Intramuscular Fat (IMF)

As IMF concentration increases, generally tenderness increases, although the relationship is frequently weak. In a New Zealand study using grass-fed lamb, higher marbled loin (marbling score was used as a proxy for IMF concentration) was more tender based on shear force than low-marbled lamb, but sensory analysis did not show any significant difference in tenderness [8]. In a large scale study (*n* = 1434) in Australia, using Meat Standards Australia (MSA) sensory protocols [9], an increase in IMF concentration in lamb loin from 2.5% to 7% increased tenderness scores by 5.9 units (on a 0–100 scale). The effect of IMF concentration on tenderness was almost half its effect on juiciness and flavour (Figure 1). The extremes in IMF concentration in this study could be sufficient to change the consumer quality grade within the MSA system. Pethick et al. (2006) [10] suggest that an IMF concentration of 4–5% for lamb should be maintained, based on reduced sensory scores when IMF concentration declines. Intramuscular fat, particularly at a higher concentration, is thought to affect tenderness through alteration of the meat structure, i.e., physical expansion of intramuscular connective tissue, thus making the muscle more tender [11]. Many of the production factors listed above can directly influence IMF concentration and, ultimately, meat tenderness.

### 2.2. Genotype

In Ireland, the predominant lowland sire breeds of the national ewe population are Suffolk (51%) and Texel (10%). The Scottish Blackface (14%) and Cheviot (11%) are the predominant sire breeds of hill ewes [12]. Both Hopkins et al. (2011) [13] and the authors of a DEFRA (Department of Environment, Food and Rural Affairs, UK) review in the United Kingdom [14] concluded that differences in meat eating quality between lamb breeds are generally small and inconsistent. In a UK study, meat from Scottish Blackface lambs was more tender than meat from Texel lambs [15], an observation confirmed in a recent Irish study [16]. Typically, variation of traits within a breed is quite large, therefore studies that conduct sensory analysis on large numbers of progeny from many sires, within and across breeds, are needed to determine the genetic parameters for eating quality of lamb [17]. Certain high muscling genotypes, such as the Callipyge and Carwell mutations, have been shown to decrease meat quality through reductions in tenderness modified, in part, by a decrease in IMF [13]. It has been suggested that the tenderness of meat (particularly of the loin and leg muscle) from sheep carrying the Callipyge mutation can be improved when post-mortem tenderisation treatments, such as electrical stimulation, prolonged aging and freezing prior to aging, are used [18]—these topics will be covered in more detail in following sections. Selecting for high muscle depth within breeds, for example, exploiting the Texel muscling QTL (Qualitative Trait Loci), increases loin muscle depth and decreases IMF concentration. Lambe et al. (2010) [19] reported that the consequent decrease in tenderness, when compared with lambs without this QTL, could be removed, provided by post-slaughter treatments, including high-voltage ES and ageing for a period in excess of 7 days. In Australia, the selection for lean meat yield through breeding for increased eye muscle depth and decreased fat depth had a negative impact on sensory scores [9]. This was in contrast to the study of Navajas et al. (2008) [15], who found similar eating quality scores of progeny from low- and high-muscling sires. However, the authors speculated that the muscularity difference between the progeny from the high- and low-muscling sires was not great enough to be detected by trained panellists. Selection for increased muscularity has also been shown to increase muscle glycogen concentration, which suggests that lambs with higher muscularity, or even different live weight [5], may have a different post-mortem pH profile that may influence tenderness (See Section 2.4).

### 2.3. Sex

Studies in several countries have shown sex to have a small and weak influence on sensory attributes, with sensory scores generally higher for meat from female animals compared with males. Pannier et al. (2014) [9] showed female lambs to have a 1.8-times higher sensory score for tenderness of the loin muscle when compared with castrated lambs (0–100 scale). Greater tenderness in muscle from females often reflects a higher IMF concentration, but this was not the case in the above study. Navajas et al. (2008) [15] found no difference in tenderness in loin or topside from ewe or ram lambs. 

Traditionally, in Ireland, male lambs were castrated mainly for ease of management. Entire ram lambs grow faster and convert feed more efficiently than their castrated counterparts [20] and, consequently, are attractive to sheep producers. Some processors and producer groups have expressed concern that using uncastrated male lambs undermines the market for lamb because consumers find the eating quality of meat from entire male lambs unsatisfactory [21]. In the UK, Dransfield et al. (1990) [22], compared the eating quality of muscle from rams, castrated rams and ewes when slaughtered at 5 months of age. There was no evidence of an effect of castration on shear force or tenderness as assessed by a trained panel. In an Irish study [23] with lambs slaughtered at 10 months of age, meat from entire males received a lower score for tenderness (3.2 on a 0–100 scale), but the overall acceptability rating for the meat from entire males was significantly better than that from castrates. In a New Zealand study [24] entire males and castrates were evaluated over a wide age range of 4 to 24 months. There was no difference in meat toughness up to age 10 months, but, at 13 months, the meat from entire males was tougher. The taste panel detected no differences in sensory evaluations up to 13 months of age, but there was evidence that some of the sensory attributes diverged when the animals were between 15 months and two years of age. Hanrahan (2010) [25] concluded, following a review of the available literature at that time, that where lambs are reared on an all-grass diet and slaughtered by the end of the grazing season, leaving male lambs entire has no negative effect on meat quality, regardless of whether assessment is laboratory-based or conducted through consumer testing. The above findings are largely supported by a recent Irish study [20] and imply that, for pasture-based production systems in which lambs are slaughtered before 12 months of age, ewes, un-castrated males and wether lambs will provide meat of similar tenderness.

### 2.4. Animal Age

It is difficult to separate the effects of age, weight and season of slaughter of lambs, since increasing age at slaughter usually increases simultaneously with carcass weight. In general, muscle from older animals is less tender than muscle from younger animals. Loin-muscle samples from older sheep, which ranged in age from 5 to 68.5 months, were less acceptable for consumers for tenderness, but there was no difference in tenderness, or shear force, in lamb or mutton from sheep slaughtered at 8.5 and 20 months of age, respectively [26]. These consumer data are supported by tenderness measured as shear force, which increased in older animals [27] likely due to decreased collagen solubility. The Pethick et al. (2005) [28] study highlighted the acceptable eating quality of the yearling sheep for this muscle. The effect of animal age varied between muscles which can, to some extent, be explained by their differing collagen concentrations and protein cross-linking with the topside having more collagen than the loin [29]. Veiseth et al. (2004) [30], concluded that lambs slaughtered at 10 months, compared with 2 months of age, were more tender and postulated this to be due to the older animals having greater subcutaneous fat and therefore greater insulation to prevent cold shortening. A similar pattern can be seen in Okeudo and Moss (2008) [6], i.e., a decrease in shear force across an age range of 6 to 12 months. In contrast, Vipond et al. (1995) [31] reported that meat from lambs slaughtered at weaning was more tender than that from lambs slaughtered post-weaning. In another study [5], it was found that lambs slaughtered at 90, 130 and 175 days of life (with respective live weights of 20, 30 and 40 kg) showed an increased shear force as age was extended. However, this trend was only observed for two muscles (*Semimembranosus* and *Biceps femoralis*). On the other hand, the *Longissimus* muscle and *Semetendinosus* were not affected by this factor.

A Meat and Livestock Commission-funded project at the University of Newcastle, UK, demonstrated an increase in toughness as lambs became older. Data are not available, and the results are only stated in Matthews and Ford (2012) [13]. “Lambs finished as hoggets (in January following spring birth) were tougher than those finished in the previous summer. Those finished in the autumn were intermediate. The possibility of this being due to some factor confounded with season of slaughter cannot be excluded—lower ambient temperature increasing the incidence of cold shortening for example”. In this respect, Miranda-De La Lama et al. (2009) [32] slaughtered Spanish light lambs of the same age (100 days) and live-weight (25 kg) in summer (ambient temperature during lairage 32.2 °C) or in winter (ambient temperature during lairage 15.6 °C). When compared with lambs slaughtered in summer, loin from lambs slaughtered in winter had higher ultimate pH (5.80 vs. 5.51) and shear force (6.60 vs. 5.53 kg/cm^2^). A further UK study concluded that “For the grass- and concentrate-fed hoggets, there was a deterioration in some quality attributes between November and March, most notably in abnormal flavour (which increased) and flavour and overall liking (which decreased). These flavour changes were more important in determining overall liking than the changes in texture and juiciness between the two slaughter times, some of which favoured the March-slaughtered groups, particularly juiciness. But it is noteworthy that all lamb groups produced meat that was tender” [33].

A recent study reported that lamb age has a little impact on eating quality when comparing new-season (240 days of life) and old-season (328 days) lambs [34]. This study was conducted in several cuts and minimal differences were observed, with higher scores for younger lambs. The lack of shear force and tenderness differences between groups lead to the hypothesis that collagen crosslinking is not evident before 12 months of age, which is one of the main contributors of toughness [34].

### 2.5. Nutrition

An increase in the energy concentration of a ration, or in the amount of the ration, can increase the IMF concentration when animals are slaughtered at the same time. Depending on the scale of the increase, this can result in an increase in tenderness (Figure 1). With respect to ration composition, the most extreme comparison is between grazed grass and concentrates offered ad libitum. The results of an study comparing grass-based with concentrate-based rations showed that the IMF concentration was higher (*p* < 0.05) in meat from sheep fed on a concentrate-based diet (4.99 g/100 g), compared with meat from sheep fed on a grass-based diet (3.39 g/100 g) [35]. Based on the minimum IMF concentration before sensory quality is impaired (4%) [10], this might indicate that concentrate-fed lamb would be more tender than grass-fed lamb. However, Australian consumers could not discriminate sensory characteristics between lambs finished on pasture and grain [26]. In Irish studies [23], no difference was found in the shear force for loin muscle when lambs were finished to a common live weight on rations that contained either 200 or 800 g concentrate/kg, offered ad libitum. Gkarane et al. (2019) [36], found no difference in the tenderness of loin muscle when lambs were finished on rations that contained grass silage only, grass silage + concentrates (50:50) or concentrates offered *ad libitum*. In a recent Australian study, consumers had a small preference for tenderness in grain-fed lamb meat compared with meat from grass-fed wether lambs, but not for female lambs [7]. When the data were corrected for IMF concentration (5.6% for grain and 4.4% for pasture fed groups), the difference disappeared. Overall, it is likely that any effect of dietary type on tenderness is small, with other associated factors such as carcass weight, IMF concentration and age, being of greater importance. 

While pre-slaughter ration composition has a minor effect on lamb tenderness, it has a bigger effect on lamb flavour. In some of the studies mentioned above, and in others, differences in overall acceptability were reported, which likely reflect differences in flavour. Consideration of the effect of pre-slaughter diet on meat flavour is outside the scope of this review. However, readers are referred to a recent comprehensive review on this topic [37].

### 2.6. Pre-Slaughter Transport/Lairage

UK studies [38,39] concluded that sheep seem to be more resistant to stress than cattle and rarely exhibit “dark cutting” conditions; i.e., high ultimate pH in muscle. It is generally accepted that handling and loading are the most stressful periods, and that the sheep will recover to some extent throughout the journey to the abattoir. Transport appears to have little stress effect on sheep and Fisher et al. (2010) [40] concluded that sheep can cope with road transport up to 48 h.

As a summary of all the pre-slaughter factors discussed in this section, Table 1 was generated based on specifications dictated in UK and Australia.

## 3. Early Post-Mortem Intervention Impact on Sheep Meat Tenderness

### 3.1. Main Early Post-Mortem Factors Affecting Meat Quality

Carcass interventions to increase meat tenderness focus on three main aspects: (i) minimising muscle contraction, i.e., maintaining the sarcomere length as long as possible; (ii) optimising the activity of proteolytic enzymes (mainly m- and µ-calpain); and (iii) standardizing the temperature at which pH 6 is reached. These are of relevance since, if early post-mortem interventions can optimise the aging effect and minimise the degree of shortening in the pre-rigor muscle, tenderness can be improved [43]. Achieving an optimum pH and temperature decline is also important, since it has been broadly reported that the temperature at which rigor is completed is a good indicator of meat tenderness [29,43,44,45,46,47].

Managing the impact of the interaction of pH and temperature in the early post-mortem phase, before the rigor has been fully resolved, has been the most studied early post-mortem intervention. The main considerations are the pH value when the carcass reaches 18 °C (pH@T18), the temperature when pH reaches a value of 6 (Temp@pH6) and pH at 24 -h post-mortem (pH24), which can be considered the ultimate pH (pHu). Hopkins et al. (2011) [48] employed these variables as predictors of shear force and showed that, for 1-day-aged product, combinations of the temp@pH6, the pH@T18 and the pH24 were better able to explain the variation observed in *Longissimus lumborum* when compared with sarcomere length alone. Besides, pH24 was the best single predictor. On the other hand, for 5-days-aged product, pH@T18 was the single best predictive parameter. It was suggested that sarcomere length can improve the prediction but is not attractive, considering the time and cost involved in its measurement. A study from the 1970’s [49] demonstrated that sarcomere length was a good predictor of tenderness when carcasses underwent a slow pH decline ratio; but the relationship was not significant when pH decline ratio was faster (<6.3 at 3 h), which frequently happens with electrically stimulated (ES) carcasses.

**Figure 2:**
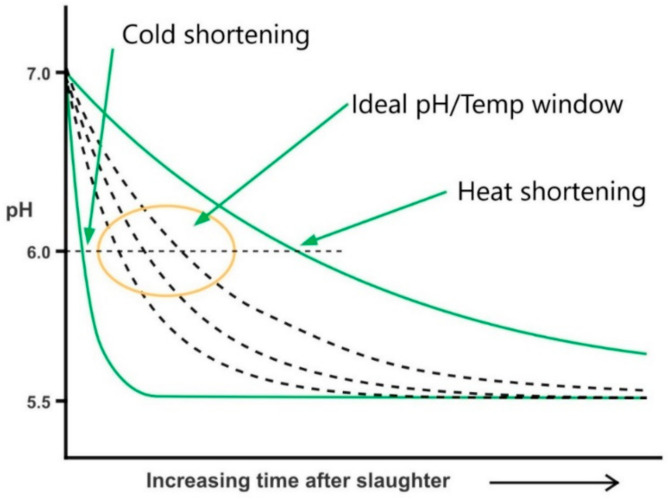
Representation of pH decline ratio over time, highlighting the regions of cold and heat shortening, and the ideal pH/temperature window to produce tender sheep meat. Adapted from [53].

Thus, proper management of pH and temperature decline can prevent the negative effects of heat and cold shortening and will give a better consistency in final product tenderness. Cold and heat shortening are causing undesirable effects on meat, which occur when the pH values decrease too quickly while the muscle temperature is still high (heat shortening), or when pH values are too high when muscle temperature declines too quickly (cold shortening), as it can be seen in Figure 2. It is generally considered that a temperature between 8 and 30 °C, when meat has a value of pH 6, is the “ideal pH decline”. After this, if followed by an aging period of 5 days, good-quality sheep meat will be generated [50]. However, this is a very broad range of temperatures and other studies suggest that 15–18 °C is the optimal temperature when the rigor has been fully resolved. For instance, a study carried out in Australia over 4 years in more than 7800 animals found that the proportion of animals achieving this ideal pH decline ranged from 0.13 to 0.69, depending on the abattoir and year; however, the large variation observed in each lot made it impossible to see significant relationships between pH declines and eating quality [46]. It has been reported that rigor mortis is completed at a constant chilling temperature of 14 °C after 12 h for non-ES animals, and around 5 h for ES animals at the same chilling temperature, as a consequence of a faster pH decline [51]. According to other authors [49], it is recommended that carcass temperature should not be lower than 11 °C while pH is higher than 6.2 units. A further challenge to achieve the desirable rate of pH and temperature decline is the influence of the weight and associated fatness of the lamb carcass. The subcutaneous fat layer acts as a form of insulation and heavy fatter carcasses have a lower rate of temperature decline, which increases the risk of heat shortening, but decrease the risk of cold shortening [52].

### 3.2. Electrical Stimulation (ES)

The values of pH in sheep can range from 6.85 to 6.05 after 45 min, according to Okeudo (1996) [54]. Consequently, achieving the right temperature (15–18 °C), at pH 6, by using a unique chilling regime, might be challenging. To achieve the above targets of pH and temperature, the strategies most employed are based on specific chilling regimes alone or in combination with ES. Specific chilling regimes (discussed in detail in Section 4) are employed with the aim of controlling muscle temperature to fall within a specific range at the time when pH 6 is predicted to be reached. On the other hand, ES provides a method to reach pH 6 at a certain temperature, while avoiding cold shortening, by accelerating the muscle acidification. However, according to MLA (Meat and Livestock Australia, Sydney, Australia) recommendations, ES is not required if sheep meat is aged for at least 10 days and if rigor has been completed within a temperature range of 8–18 °C. The use of ES has the potential to standardize pH values when applied early after slaughtering, making a common chilling regime more feasible for carcasses treated in this way. For these reasons, the most common practice is to combine specific chilling regimes with ES. Hwang et al. (2003) [55] reported that ES can be a tool to increase glycogen metabolism with the concomitant faster accumulation of lactic acid and, therefore, leading to lower pH value in muscle when compared with unstimulated muscles. Faster pH declines will allow applying increased chilling regime speeds, which will lead to a reduction of the evaporative loss in the carcass and prevents microbial growth. However, some carcasses have a natural fast rate of pH decline and this, along with ES, can cause a heat-shortening effect, whereby the pH value drops faster than expected and the meat temperature is still high when reaching pH 6. 

It is still not very clear what exact mechanisms underpin the increase in tenderness after ES, even if the same pH/temperature profile is observed. According to Devine et al. (2006) [51], when rigor mortis occurs at the same temperature, ES has a positive effect on tenderisation. However, this experiment was carried out using individual muscles that were hot boned and then tightly wrapped, following an immersion in cold water to standardize muscle temperature over time. Three main modes of action were proposed [55] by which ES enhances meat tenderness. (1) The prevention of cold shortening by ensuring that pH/temperature declines are optimum or at least outside the cold-shortening window; (2) a physical degradation of the muscle fibre; (3) and an acceleration of proteolysis in the aging period. It was also found [51] that animals resting from farm handling for a longer period of time (eight or ten days, compared with one or three days) were able to reach a lower pHu, although this was ultimately unrelated to a difference in tenderness after 72 h of ageing. It was also observed that, within the same range of pHu, a higher number of ES animals were found to have tender meat. Under controlled aging conditions (3 days at 15 °C), intermediate pHu values were enough to achieve tender meat in almost all animals, whereas, at shorter aging times (4 and 24 h), a greater difference in shear force was observed between ES and non-ES animals. 

Electrical stimulation is often used prior to rapid chilling and has been demonstrated to improve tenderness in both objectively (Warner–Bratzler shear force determination) and subjectively (consumer sensory scores) for carcasses hung in the conventional manner (Achilles tendon) [56]. Sheridan et al. (1998) [57] reported that electrically stimulated carcasses initially showed greater tenderness compared with non-electrically stimulated carcasses (as determined by six trained panellists) after 24 h, but further ageing to 5 days eliminated this effect. 

As previously mentioned, a combination of ES and chilling regimes is an interesting tool to control the temperature at which rigor is completed. Thompson et al. (2005) [43], studied the interaction between ES, chilling rate, carcass suspension, aging time and animal characteristics on meat eating quality. A study carried out in an Australian abattoir reported that the number of carcasses following the ideal pH/temperature window decline with ES was 60%. Meanwhile, around 85% of non-ES carcasses had a pH higher than 6 when reaching 18 °C. Age category (lamb or mutton) had the most significant effect on tenderness, followed by muscle type, ageing and carcass suspension method. However, chilling rate and ES were found to be useful just to manipulate temp@pH6, but they were not considered a treatment *per se*, since it was not possible to standardize both interventions and no fixed effect could be attributed. 

When analysing the temperature at which pH 6 was reached, ES was the most relevant factor, followed by chilling rate and slaughter group (day and abattoir). For this reason, it is essential to ensure that outcomes from ES are carefully monitored to ensure the desired effect on pH decline in combination with the adequate chilling regime. The impact of temp@pH6 can be observed in the next figure. As can be seen, if just temp@pH6 is considered, a temperature around 20 °C is the most adequate; unless tender stretching (i.e., by hanging them from a hook placed under the pubic symphysis) is considered; in this case, very fast or very slow chilling regimes are recommended for optimum tenderness.

### 3.3. Hanging Method

To prevent the detrimental effect of heat shortening, muscle stretching can be carried out, since it limits the overlapping between actin and myosin that occurs when the muscle contracts. Warner et al. (2014) [58] examined the efficacy of carcass-hanging methods to prevent the impact of heat shortening in lamb. For this purpose, lambs were hung using two different methods (stretching of a carcass by tying the leg to the ribs and conventionally, i.e., by the *Achilles* tendon), and then exposed to two different pre-rigor temperatures (37 and 2 °C):

It was concluded that the stretch treatment resulted in longer sarcomere length and less contraction in those muscles actually affected by the hanging method (*Gluteus medius* (GM), *semimembranosus* (SM) and *semitendinosus* (ST)), resulting in a significant improvement in tenderness, even at high rigor temperatures. Further experiments were performed to elucidate the impact of duration of exposure of high pre-rigor temperature (1.5, 3 and 4 h) combined with different hanging methods [59]. The main conclusion was that a longer time at high temperatures had a negative effect on the quality traits of the meat; however, muscle stretching prevented the negative effect on shear force, although other factors such as cook loss could not been prevented.

MLA has a specific requirement when combining aging, hanging methods and ES to comply with MSA (Meat Standards Australia) [41]. These are shown in Table 2.

### 3.4. Post-Mortem Aging

Although aging is not strictly an early post-mortem intervention, the events taking place in the first 24 h after killing may have an impact on the subsequent aging or tenderisation. At this stage, endogenous proteolytic enzymes degrade specific structural proteins, leading to a loss of the myofibrillar structure and, therefore, more tender meat. It has been reported for lamb that, when using WBSF method, a 49.0-N threshold is the upper limit for consumer acceptance [60]; in a more recent study it was found that tenderness values lower than 40.0 N were classified as “good everyday-quality” by a consumer panel [61].

Li et al. (2019) [62] highlighted that tenderness is mainly influenced by sarcomere length and an early freezing processes can accelerate the ageing rate of lamb loins. In their study, lamb carcasses were aged for different times (0 to 48 h) at 4 °C and then frozen for three weeks at −20 °C. After this, the lambs were thawed and the ageing time was balanced, up to a total time at 4 degrees for 120 h. It was observed that pH was stable 24 h after slaughter. However, it was noticed that pH was significantly lower for those carcasses that had been frozen, probably due to a higher loss of moisture after thawing. It was concluded that the earlier the lamb loins were frozen the more tender the meat, coinciding with a higher degradation of structural proteins (desmin and troponin). However, cook loss was markedly increased in the treated samples. It was recommended that freezing should take place 12–24 h after killing.

Two recent investigations [63,64] concluded that ageing lamb beyond 7 days does not significantly decrease shear force. According to Dransfield (1990) [22], 80% of the tenderisation of lamb *longissimus dorsi* occurs by 7.7 days post slaughter at 1 °C. MLC (Meat and Livestock Australian Commission) trials showed a benefit of ageing lamb loin for up to 10 days, with most of the benefit occurring by 7 days [50]. In another study [5], tenderness in four ram lamb muscles was compared after 24 h and 7 days of aging. The authors reported an increment in tenderness for all the muscles but for semitendinosus muscle, regardless the age or living weight of the animal at slaughter. A recent study [65] highlighted that after 14 days of aging an improvement in tenderness was achieved for the *longissimus lumborum* and *gluteus medius* muscles; however, no samples were analysed at an intermediate aging time, so it is not possible to conclude when the optimum aging time was reached. 

The difference found in tenderness [66] was much higher when comparing non-ES and ES carcasses than when aging time was extended from 5 to 10 days. The interaction between carcass management protocol and aging time was significant for all sensory traits assessed (texture, juiciness, lamb flavour and abnormal flavour); the best results were obtained after ES and 10 days of maturation. It was very interesting that non-ES and 10days-aged lamb was less tender than ES and 5-days-aged lamb meat.

The role of internal proteolytic enzymes (m and µ-calpain), along with their inhibitor (calpastatin), has been a matter of intense research in recent years. An excellent review has been recently published [67]. It was stated that the rate of pH decline, at the early post-mortem phase, has a significant impact on the activation of µ-calpain autolysis, which is suggested to have the main role, compared with m-calpain, in the tenderisation process during aging. However, a fast pH decline also promotes accelerated proteolysis in the calpain substrates (i.e., structural muscle protein), enhancing final tenderness. m-calpain is still active in lamb muscles after 56 days of maturation at 30–40% of its initial activity measured [68]; however, µ-calpain activity is lost within the first 7 of aging, due to inactivation and autolysis (at least in beef, since no equivalent information was found on sheep) [69,70], although a residual activity can be detected even after long aging times in lamb [68]. The effect of temperature on calpain activity during the onset of rigor was negligible at pH values higher than 6.2; below these pH values, calpain activity decreased [71]. Under these conditions (low pH and high temperature) contractile proteins are more stable and calpain autolysis is more evident, which are undesirable; therefore, the aging process is not as effective and the potential for tenderisation is reduced [72].

Since µ-calpain needs to be activated by autolysis, it was observed that the velocity of activation of this enzyme in lamb meat incubated at high temperatures (25 to 35 °C) was increased [45]. It resulted in higher degradation of meat proteins in the early stages. However, due to the further extent of µ-calpain autolysis, there was a remarkable loss of activity over the ageing period and a decrease in the degree of myofibrillar degradation, leading to poor-quality meat. According to this research, an optimum ageing temperature, when rigor is established, is approximately 15 °C. In this way, increased tenderness can be achieved while WHC (water-holding capacity) is not affected, but it may have a negative impact on microbial quality, as described in detail in the next section. It was also found [62] that degradation of structural proteins (desmin and troponin T) was faster after freezing. Probably, the formation of ice crystals destroyed the sarcoplasmatic reticulum, releasing calcium and activating u-calpain; although deactivation of calpastatin, due to freezing, might play a role. 

The activity of proteolytic enzymes can also be modulated by pre-slaughter interventions. For instance, it was shown that energy intake restriction will have a negative effect on the activity of calpastatin, thus modifying the calpain/calpastatin balance [73]. As a consequence, a positive impact was observed in the final tenderness of meat lamb, since a very strong correlation (r = −0.64) was found between calpain/calpastatin ratio and the change in shear force over aging time. In a paper published by della Malva et al. (2017) [74] it was reported that a diet rich in omega 3 fatty acids might have a protective effect against stress, minimising the pre-slaughter glycogen breakdown. Consequently, a low pH value in the first 3 h post-slaughter was prevented, since initial pH of muscle was higher. However, it had no effect on pHu. Even more, diet can alter the pattern of myofibrillar proteins oxidation, altering their susceptibility to hydrolysis during the aging time, and, therefore, having a negative impact on final tenderness [75]. A positive relationship between the number of myosin light-chain isoforms and tenderness was found and the number of isoforms was related to the diet, with linseed being the most favourable dietary supplement.

Finally, a recent topic of research attracting attention is the impact of apoptotic factors in the activity of calpain and calpastatin. Molecules such as cytochrome C and caspases have been related with sheep meat tenderness and the activation of the calpain/calpastatin system, since an overexpression of these factors leads to tender meat [76]. On the other hand, heat shock proteins (HSP), when overexpressed, were associated with an increment in sheep toughness. However, the apoptosis mechanism, which is generally considered to start immediately after exsanguination, is a genetic factor and therefore is unlikely to be controlled by means of a carcass intervention. However, since these proteins are massively produced in stressful situations, such as extreme heat or cold, ischemia, trauma, exercise or diseases, a controlled environment before slaughtering will help to reduce the over expression of HSP proteins. Low-stress conditions will promote normal tenderisation. The fact that excessive stress depletes glycogen, impeding a regular pH decline in meat, along with the overexpression of HSP, could have a synergetic effect on decreasing final meat quality [77].

### 3.5. Chilling Regimes

Chilling rate can have a positive impact for both the consumer and industry, as it influences the visual appearance and eating quality characteristics of meat, as well as the evaporative loss and rate of throughput of carcasses in a slaughter plant [78]. Temperature, relative humidity, air velocity and time are the four variables that define a chilling rate [79]. Finding the ideal chilling rate that minimises toughening of meat and microbial proliferation of spoilage organisms while maximising shelf life is a critical challenge for industry [80]. McGeehin et al. (2001) [81] conducted a study to elucidate the factors that might affect the pH value recorded at different post-mortem times using a single chilling regime of 4 °C for 24 h. In this Irish study, which lasted one year, it was shown that sex, carcass weight, and, with less relevance, age and ambient temperature affected pH values; however, seasonality had a negligible effect. This research showed that pH at 4 h and at 45 min (R) can be estimated (R^2^ = 0.74) based on the above-mentioned factors. This will give an indication of the most adequate chilling regime to be employed to reach pH 6.0 at 18 °C. Aggressive chilling regimes will have a negative effect on the kinetics of glycolytic-related enzymes, thus, the pH decline will be slower; however, it is still controversial (based on the literature consulted) whether the temperature itself can modify the pH-decline ratio in a way that can finally affect the meat quality. McGeehin et al. (2002) [79] tried to optimise an ultra-fast chilling regime for improved lamb meat quality. In this research several combinations of very low air temperatures (−10 to −25 °C), a chilling duration of 2.5 or 3.5 h and two wind speeds (0.5 and 1.5 m/s) were tested. After this period, the carcasses were moved to a chilling regime at 4 °C. It was found that pH values were not significantly different at 1 and 4 h post-mortem when compared with a conventional chilling regime (4 °C for 24 h). More interestingly, although no specific relationship with any of the chilling parameters could be established, pH at 24 h was found to be slightly, although significantly higher, in some of the ultra-fast chilling regimes. When tenderness was evaluated, 8 of the 12 regimes were tougher than the conventional chilling regime after one day of ageing, and this number was reduced to six after 5 days of aging. The main factor affecting shear force was the chilling duration, where 3.5 h of treatment yielded an average value of 55 N in contrast with 45 N when only chilled for 2.5 h. Similar results were obtained from their sensory analysis, wherein only three of the treatments were scored significantly poorer than the control. The same research group further confirmed that pH and shear force were unaffected by chilling conditions [68] in Irish lambs.

### 3.6. Other Interventions

Although chilling regimes and ES are the most common interventions employed at the industrial level, many other technologies have been tested with different efficacies in terms of tenderness improvement. A recent review [82] discussed in detail the various available methods to improve meat tenderness in the early post-mortem stage. Among the most relevant technologies are those based on high-pressure processing (HPP), pulsed electric fields, shockwaves, infusion, ultrasound, Smartstretch^®^ or PiVac^®^, which are based on a hot-boning process followed by wrapping the primals in a flexible material to prevent muscle contraction. Using a meta-analysis approach, it was found that HPP, followed by Smartstretch^®^, achieved the highest reduction in peak shear force, of 43.5 N and 10.8 N, respectively [82]. However, implementing such technologies at the industrial scale can be challenging in terms of cost, implementation and optimisation.

The use of chilled solution (14 °C, 98.5% water and the balance of glucose, polyphosphate and maltose) infused into the vascular system, immediately after slaughtering, has also been found to improve the tenderness of lamb. This technology, named Rinse and Chill^®^ (RC), facilitates carcass chilling and faster blood removal. A recent study investigated the use of combined ES and RC to improve lamb meat tenderness [83]. It was concluded that ES followed by RC could reduce the number of carcasses exhibiting cold shortening.

## 4. Chilling Regimes and Their Impact on Meat Quality and Microbiological Status

Until recently in the European Union, Regulation EC 853/2004 [84] was in place, which mandated that carcasses must be immediately chilled after post-mortem inspection to ensure a temperature of not more than 7 °C, in the case of meat, and not more than 3 °C for offal. However, neither the time limit by which this was to be achieved nor the reasoning behind the selection of these target temperatures was ever specified by the regulation. In response, the European Food Safety Authority (EFSA) Panel on Biological Hazards (BIOHAZ was asked to issue a scientific opinion on the public health risks associated with applying flexibility in the cold chain during storage and transport of meat. It considered research studies conducted in the area, as well as the modelling of the growth of key pathogens (*Salmonella* spp., *E. coli*, *Listeria monocytogenes* and *Yersinia enterocolitica*) on the surface of beef and pork carcasses using hypothetical chilling curves [85]. The scientific opinion concluded that the surface temperature of the carcass, rather than the core temperature, was a more relevant indicator of the effect of chilling on bacterial growth, and that alternative time-temperature chilling profiles resulting in equivalent or less bacterial growth than that obtained under Regulation EC 853/2004 could be used in beef, pork and sheep slaughterhouse chillers. A further scientific opinion [86], addressed the issue of the growth of spoilage bacteria during storage and transport of meat using the same hypothetical chilling curves used to model pathogenic growth in the previous study. The opinion found that some spoilage bacteria, in particular *Pseudomonas* spp. and lactic acid bacteria, can reach critical levels more quickly than pathogens and this is dependent on both the initial contamination levels and the temperature conditions under which the carcasses are stored. With this in mind, the European Commission published Regulation EU on the 31 October 2017 [87], which amended Regulation EC 853/2004. This allowed for the introduction of alternative temperature conditions during transport of fresh meat, in particular carcasses or larger cuts without any increased public health risk and without deviating from the basic principle that such meat should be chilled to 7 °C by a continuous decrease of temperature.

Many studies have been conducted to determine the optimum chilling rate of carcasses in chillers, with the focus mainly on beef, and a few studies on other meat types, such as sheep. The next section will discuss, in more detail, the various chilling parameters that have been studied in sheep, with a focus on the changes in the microbiology status of the carcass.

### 4.1. Microbiology of Sheep

Fresh meat is prone to microbial spoilage due to the high water activity (>0.99), ideal pH (>5) and nutrients present in the product that enable microbes to grow. Indicator organisms, such as TVC and total *Enterobacteriaceae* counts (TEC), are regularly used in industry to determine the hygiene characteristics of carcasses. The European Commission Regulation 1441/2007 [88] established satisfactory microbiological criteria for sheep carcasses before chilling with TVC’s not to exceed 5 log_10_ cfu/cm^2^ (colony-forming units per centimetre squared) and TEC’s not to exceed 2.5 log_10_ cfu/cm^2^. If these limits are exceeded, then the abattoirs are required to take certain action, including improvements in slaughter hygiene and a review of process controls. While the slaughter process, particularly hide removal, is an important source for this microbial contamination of carcasses, other sources, such as aerial contamination during chill storage, have been identified [76]. Although sensory changes in meat, such as colour and odour, are the most important factors to consumers when purchasing meat, these are largely linked to the microbial contamination present on the meat, with the shelf life of refrigerated sheep meat usually exceeding 10 days before the first signs of spoilage are noticed [89]. The industry considers refrigerated meat to be spoilt when the TVC population reaches 7 log_10_ cfu/cm^2^, but the reasoning behind this baseline figure is unclear, particularly when studies have shown that the visual colour and odour of vacuum-packed lamb shoulders remains acceptable when TVC’s increased to over 8 log_10_ cfu/cm^2^ [90]. This is one of the drawbacks of using total microbial populations as a determination of shelf life rather than individual communities, particularly as the type of community influences the amount of volatile organic compounds produced by bacterial metabolism. 

During aerobic storage (carcass chilling), *Pseudomonas* spp. are the primary organisms that grow on sheep meat with *P. fragi*, *P. lundensis and P. fluorescens* the three main species related to spoilage. Their psychrotrophy, very fast growth rate and high affinity for oxygen have been suggested as the main reason for their predominant growth on carcasses, leading to a rapid uptake of glucose, the first substrate used by most bacteria during chill storage [91]. When glucose is depleted, *Pseudomonas spp*. can attack amino acids, with the consequent production of malodourous sulphides, amines and esters [92]. Under low concentrations of glucose under aerobic storage, spoilage-related lactic acid bacteria, such as *Carnobacterium* spp. and *Lactobacillus* spp., may oxidise lactate and its catabolic precursor, pyruvate, into acetate. The increase in acetate may have a negative impact on the sensory quality of meat, due to its sharper vinegar-like flavour [83]. Under anaerobic storage (i.e., vacuum packed or a modified atmosphere), lactic acid bacteria and *Brochothrix thermospacta* become the predominant spoilage organisms, due to their ability to grow in the presence or absence of oxygen. *Pseudomonas* spp. remains a persistent spoiler, due to the low levels of oxygen permeating through the packaging, but they grow slowly and their levels remain much lower than the anaerobic bacteria [93]. *Carnobacterium* spp. and *B. thermospacta* are the major producers of volatile fatty acids such as butanoic acid, which are major causes of meat spoilage, resulting in rancid/buttery flavour and odour [92]. Psychrotrophic, anaerobic clostridia are also major meat spoilers and break down glucose to form butyric acid, butanol, carbon dioxide and hydrogen, resulting in a discoloration of the meat. While yeasts and moulds are also present on meat, they are rarely monitored as spoilers, as they are mainly detected after a long shelf life or when meat is stored aerobically and has a low water activity [94].

While storage atmosphere, pH and water activity (aw) all influence bacterial population dynamics, the most important factor is the storage temperature of the meat. Carcasses held under conventional chilling and delayed chilling all promote the proliferation of bacteria, as the surface temperature of the carcass does not drop to a temperature that is capable of reducing bacterial growth for a few hours during chill storage. On the other hand, rapid chilling reduces the surface temperature more quickly and, therefore, there is reduced microbial contamination on the surface of the carcass. Balancing cooling temperature to minimise microbial contamination and carcass weight loss and to maximise tenderness and eating quality remains the key target for meat science and shelf-life studies.

### 4.2. Conventional Chilling

In Ireland, lambs are generally chilled between 0–4 °C for 16 h with low wind speeds (~0.2 m/s) and 90% relative humidity [79]. These parameters result in the internal loin (*M. longissimus thoracis*) temperature of lamb carcasses reaching 14 °C in 5 h [68]. While conventional chilling allows the lamb core temperature to reach 7 °C after 16 h, in addition to controlling factors that promote or inhibit cold shortening during storage, it also has a number of drawbacks. Given the 16-h chilling cycle, this usually means that lamb carcasses are kept overnight in chillers, which affects product turnover in factories. Depending on the size of the plant and the number of carcasses processed in a given day, the overnight storage time means that larger chill rooms are required for carcasses than if a plant operated a ‘day-in, day-out’ procedure [95]. As fresh sheep has a relatively short shelf life, the elimination of overnight storage would be beneficial to the product shelf life. Conventional chilling also results in high evaporative weight loss in lamb carcasses, due to exposed musculature and high surface-to-volume ratio [96]. Studies have reported that evaporative weight losses can range from between 1.5% to 3% in conventionally chilled lamb [79,97], which is a major economic disadvantage for the industry. There appears to be a relationship between the chilling temperature and the initial hot carcass weight, as heavy carcasses (≥12 kg) have the most subcutaneous fat thickness, which lessens moisture losses by reducing the effective moisture vapour transmission rate from the exterior of the muscle to the surrounding air currents [98]. Finally, the delay in reducing the surface temperature of the carcass to below 5 °C allows microorganisms to proliferate, resulting in higher populations than if a lower chilling rate were applied. On lamb carcasses, the brisket is the most contaminated area and the neck is the least [99].

### 4.3. Rapid Chilling

There is no clear consensus on the nomenclature to describe chilling systems that can reduce the temperature of the carcass as quickly as possible. Terms used include ‘rapid chilling’, ‘very fast chilling’, ‘ultra-rapid chilling’ and ‘blast chilling,’ and all consist of a two-step process characterised by a first stage after slaughtering of low temperatures (usually below 0 °C) and rapid air movement (≥3.5 m/s) for a short duration (~3.5 h), and then conventional chilling for the remainder of the chilling process. For the purpose of this review, rapid chilling will be the term used to describe this type of chilling system. Rapid chilling causes the surface temperature of the carcass muscle to drop to between 0 °C and −1 °C within 5 h [78], which is faster than conventional chilling and is capable of reducing bacterial growth. Fernández and Viera (2012) [100] reported significantly lower aerobic TVC counts on rapidly chilled carcasses (−20 °C for 3.5 h with 2 m/s wind speed) than carcasses from conventionally chilled treatments, after 24 h. Furthermore, Liang et al. [101] used chill regimes of −20 °C and −35 °C air temperatures with wind speeds of 3 m/s and observed decreases in populations of psychrotrophs and *Corynebacterium,* resulting in an increased diversity of microbiota and reduced carbon and purine metabolism. 

Another benefit from applying rapid chilling with carcasses is the reduction in evaporative weight loss, which is significantly lower than in conventional chilling. McGeehin et al. (1999) [102] reported a weight loss of 0.57% compared with the 1.48% for conventional chilling. Although other authors [91] reported a higher weight loss, of 1.57%, in their study, this was balanced by the higher weight loss in their conventional treatment (2.7%). Unlike conventional chilling, humidity is a factor that cannot be controlled in rapid chilling. The reduced weight loss in carcasses may be due to the higher humidity in the second stage of chilling, when carcasses have been rapidly chilled and are stored conventionally for the remainder of the chill process. Although most of the weight loss occurs in the first phase of chilling, condensation may form on the carcasses, which would cause them to gain weight. When rapid-chilling regimes are employed, a cold shortening effect can be observed [95]; however, this is not enough to explain the loss in tenderness observed. An experiment that investigated the effect of different internal muscle temperatures at 4 h post mortem [103] revealed that meat that underwent the lowest chilling temperature experienced the highest shortening effect; however, sensory score and proteolysis degree were found to be the highest under this chilling regime. This was related to a higher pH and a remarkable increase in the calcium level, which lead to calpain activation and therefore a more effective proteolysis during the aging period. In terms of tenderness and eating quality, there are mixed reports on the effect of rapid chilling. When rapid chilling and conventional chilling are compared, rapid chilling appears to result in lower sarcomere lengths and higher shear force values [57,78,102]. Taking these data on their own, this implies that rapid chilling resulted in cold shortening, causing toughness in meat. However, when instrumental data are combined with sensory analysis, trained panellists were unable to detect differences in tenderness from meat between conventionally chilled and rapidly chilled carcasses, regardless of the sarcomere length and shear force values. It might be an indication that sarcomere length alone is not always a good indicator for tenderness, as also concluded in another report [63], where IMF concentration and desmin degradation were also relevant. Interestingly, when pH was recorded under the different chilling regimes at 1, 4 and 24 h post mortem, no differences were observed [81], in spite of the carcass temperature being significantly different at 4 h, which may affect the kinetics of glycolytic enzymes [45]. Using six experienced panellists that measured tenderness on a hedonic scale, it was reported that there was no significant difference in tenderness between samples from conventionally chilled carcasses and those that underwent rapid chilling (−20 °C with air speed 1.5 m/s for 3.5 h), or even an intermediate chill regime (−2 °C with an air speed of 2.5 m/s for 24 h) [81]. A follow-up study by the same team [79] used 10 rapid-chill regimes combining different temperatures, air speeds and chill durations, and samples underwent sensory analysis using six trained panellists that evaluated the samples based on tenderness, juiciness, chewiness and overall acceptability. Chill duration proved to be an important factor in this study, with noticeable differences in sensory parameters detected in carcasses undergoing rapid chill for 3.5 h when compared with conventionally chilled samples. It was recommended that a chilling duration of 2.5 h would balance weight loss and tenderness. Although rapid chilling increases a plant’s capital and operational costs, particularly if ES is also required, this cost can be balanced by the increased factory revenue garnered due to lower carcass weight loss [73].

### 4.4. Other Chilling Regimes

Most chilling regimes operate on the principle of forced convection air chilling, in which cold air is produced by evaporator coils positioned above the chill room. Other carcass chilling options are available for use by industry, each with benefits and drawbacks. Spray chilling, for example, operates on the same principle as air chilling except that it uses potable water as a fine spray to cool the carcass. Rather than a continuous spray, it operates intermittently, spraying at 5 and 15 min after the start of air chilling, and repeated every four or five occasions for between 3 and 8 h post slaughter, depending on carcass type [76]. While spray chilling does not appear to provide any benefits to the colour or tenderness of the meat, its main benefit is reducing evaporative weight loss in carcasses, as the water applied replaces water lost through evaporation and therefore maintains a wet surface [95]. There is evidence that this advantage may be lost further down the sheep chain, due to increased purge loss [96]. Although spray chilling reduces surface temperature more quickly than air chilling [76], a wet surface should, theoretically, support the growth of microorganisms. Brown et al. (1993) [104] monitored TVC populations on the breasts, legs and diaphragms of lamb carcasses that were spray chilled or chilled conventionally. While there was no difference in microbial populations on the breasts and legs, there was significantly higher TVC’s on the diaphragms of spray chilled carcasses, suggesting that spraying water on the carcass simply displaces the microbial population to the lower half of the carcass. In addition, recirculation of potable water is permitted in the United States and other territories but is not permitted in the European Union. This results in increased plant capital and operational costs when spray chilling is applied to carcasses.

Delay chilling is another alternative for the industry and has been suggested to cause a positive influence on post-mortem tenderness [96]. The process involves keeping carcasses out of the chill room for a length of time (approximately 7 h) and storing them at 12–15 °C until rigor has set in. Storing carcasses at these temperatures has the greatest benefit for meat tenderness, as it allows the pH to naturally drop to ~6.0 while the carcass temperature is between 10–15 °C [105]. Delay chilling did have a positive impact on the instrumental tenderness results with higher sarcomere lengths and lower shear force values compared with conventional and ultra-rapid-chill carcasses, and this was reflected in the scores given by eight trained panellists. As such, delay chilling is only recommended where the industry would prefer meat to be more tender rather than have a long shelf life.

Carcasses can be subsequently chilled using a conventional or rapid-chill approach. In a sense, delay chilling could be viewed as a cheaper alternative to ES. Fernández and Vieira (2012) [100] reported the effects of delay chilling on lamb meat as regards the microbiological and sensory characteristics of the meat during storage. They reported that carcasses had higher TVC populations on delay-treated carcasses compared with conventionally chilled and rapidly chilled carcasses. This makes sense as storing carcasses at a high temperature (above 10 °C) would create an ideal environment for bacteria to multiply. While the TVC populations did not exceed the limits set out by Regulation 1441/2007 [79], the high bacterial counts would have a negative impact on the shelf life of the final product. In addition, the evaporative weight loss was similar to conventionally chilled carcasses (2.2%).

## 5. Conclusions

Sheep meat production systems represent the combined and interactive effects of the pre-slaughter and post-mortem factors mentioned above. Meeting the carcass weight and carcass fat specifications of particular markets seems to be the most important strategy for ensuring tenderness of sheep meat. Given the relatively weak relationship between carcass fat score and IMF concentration, a method to measure IMF concentration in the live animal would facilitate more appropriate selection of animals for slaughter.

Carcass interventions should be focused on a few key points: (a) achieving a meat pH value of 6 when temperature is within a range of 8 and 30 °C (ideally narrowed to 15–18 °C); (b) ES helps to modulate pH decline, and should be used in combination with rapid-chilling regimes; (c) short aging times (5 days) are recommended ES-treated animals; (d) however, the effect of ES is negligible if sheep is aged for 10 days, having, rather, the most benefit over the first 7 days; and (e) hanging method has a positive impact on those muscles actually stretched, but may be detrimental for other muscles, which suffer contraction.

The most suitable chill regime depends on whether the industry wishes to focus on increased tenderness, improved shelf life or reduced evaporative loss. Delay chilling has the most beneficial effect on meat tenderness, but at the cost of increased evaporative loss and higher microbial counts compared with other chill regimes. Rapid chilling may be the best alternative chill regime, as it results in higher carcass throughput, reduced microbial counts and evaporative weight loss.

## Figures and Tables

**Table 1:** Pre-slaughter specifications for assuring the tenderness of lamb meat.

Factor	United Kingdom [41]	Australia [42]
genotype	little effect, not specified	not specified (Merino) ^1^
sex	rams 5–6 months ^2^	not specified
age	not specified, older animals tougher	lambs better than hoggets?
diet	little effect, not specified	little effect, not specified
growth rate	consistent moderate	100–150 g/day for at least 2 weeks pre-slaughter
transport	minimise stress, avoid mixing of unfamiliar groups	time off feed <48 h, minimise stress
lairage	not specified	<24 h
carcass	fat class 2–3 (EUROP)	fat score 2

^1^ Due to concerns of off-flavouring, ^2^ the Merino breed is more stress-sensitive than other breeds.

**Table 2:** MLA recommendations for aging time depending on hanging method and use of ES on lambs. Adapted from [54].

Ageing Period	Hanging System	Required Temp@pH6 (°C)
short ageing period of 5 days	Achilles hung	18–35
short ageing period of 5 days	tender stretch/pelvic hung	8–35
longer ageing period of 10 days	Achilles hung	8–18
